# Gene expression profiling of periodontitis-affected gingival tissue by spatial transcriptomics

**DOI:** 10.1038/s41598-018-27627-3

**Published:** 2018-06-19

**Authors:** Anna Lundmark, Natalija Gerasimcik, Tove Båge, Anders Jemt, Annelie Mollbrink, Fredrik Salmén, Joakim Lundeberg, Tülay Yucel-Lindberg

**Affiliations:** 1 Department of Dental Medicine, Division of Periodontology, Karolinska Institutet, Huddinge, Sweden; 2 Science for Life Laboratory, Department of Molecular Medicine and Surgery, Karolinska Institutet, Solna, Sweden; 3 Science for Life Laboratory, Department of Medical Biochemistry and Biophysics, Karolinska Institutet, Solna, Sweden; 4 Science for Life Laboratory, Department of Gene Technology, KTH Royal Institute of Technology, Stockholm, Sweden; 5 Hubrecht Institute-KNAW (Royal Netherlands Academy of Arts and Sciences) and University Medical Centre Utrecht, Cancer Genomics Netherlands, Utrecht, The Netherlands

## Abstract

Periodontitis is a highly prevalent chronic inflammatory disease of the periodontium, leading ultimately to tooth loss. In order to characterize the gene expression of periodontitis-affected gingival tissue, we have here simultaneously quantified and localized gene expression in periodontal tissue using spatial transcriptomics, combining RNA sequencing with histological analysis. Our analyses revealed distinct clusters of gene expression, which were identified to correspond to epithelium, inflamed areas of connective tissue, and non-inflamed areas of connective tissue. Moreover, 92 genes were identified as significantly up-regulated in inflamed areas of the gingival connective tissue compared to non-inflamed tissue. Among these, immunoglobulin lambda-like polypeptide 5 (*IGLL5*), signal sequence receptor subunit 4 (*SSR4*), marginal zone B and B1 cell specific protein (*MZB1*), and X-box binding protein 1 (*XBP1*) were the four most highly up-regulated genes. These genes were also verified as significantly higher expressed in gingival tissue of patients with periodontitis compared to healthy controls, using reverse transcription quantitative polymerase chain reaction. Moreover, the protein expressions of up-regulated genes were verified in gingival biopsies by immunohistochemistry. In summary, in this study, we report distinct gene expression signatures within periodontitis-affected gingival tissue, as well as specific genes that are up-regulated in inflamed areas compared to non-inflamed areas of gingival tissue. The results obtained from this study may add novel information on the genes and cell types contributing to pathogenesis of the chronic inflammatory disease periodontitis.

## Introduction

Periodontitis is a chronic inflammatory disease that affects tissue and bone structures surrounding the teeth. The progressive destruction of tooth-supporting tissue and bone, which may eventually lead to tooth loss, is the hallmark of periodontitis^1^. The disease is highly prevalent and approximately 46% of the adult population is estimated to suffer from mild, moderate, or severe forms of periodontitis^2^. In addition, periodontal disease has been linked to increased risk for systemic diseases, such as cardiovascular disease, rheumatoid arthritis, diabetes, and cancer^3–9^. However, the specific genes, cells or cellular mechanisms behind the pathogenesis of periodontitis are not yet known and there are today no pre-diagnostic markers or therapeutic targets for the disease.

Periodontal disease is initiated by a biofilm forming in connection to the gingiva, and its release of substances such as lipopolysaccharides and toxins. This process elicits a local immune response with subsequent stimulation of various cells and production of pro-inflammatory mediators, including chemokines, cytokines, matrix metalloproteinases, and prostaglandins, which leads to tissue and bone destruction^10–12^. A few studies have reported on gene expression in gingival tissue from patients with periodontitis, using microarrays or RNA sequencing (RNA-seq)^13–17^. These studies have revealed differences in gene expression between periodontitis-affected and healthy tissue, where gene expression related to inflammation, apoptosis, and cell death are strongly represented in periodontitis-affected tissue^13–17^.

However, a drawback with standard RNA-seq used in the above-mentioned studies is that the experiments are performed on whole biopsies and therefore produce an average of the gene expressions of all cells present in the tissue. Thus, it is not possible to localize and associate gene expression with corresponding cells in the tissue. Therefore, the aim of this study was to analyze the gene expression in periodontitis-affected gingival tissue using a novel spatial transcriptomic sequencing method that allows for analysis of gene expression in a tissue section, simultaneously associating gene expression with specific cell types.

## Results

### Gene activity in different compartments of the gingival tissue

**Figure 1:**
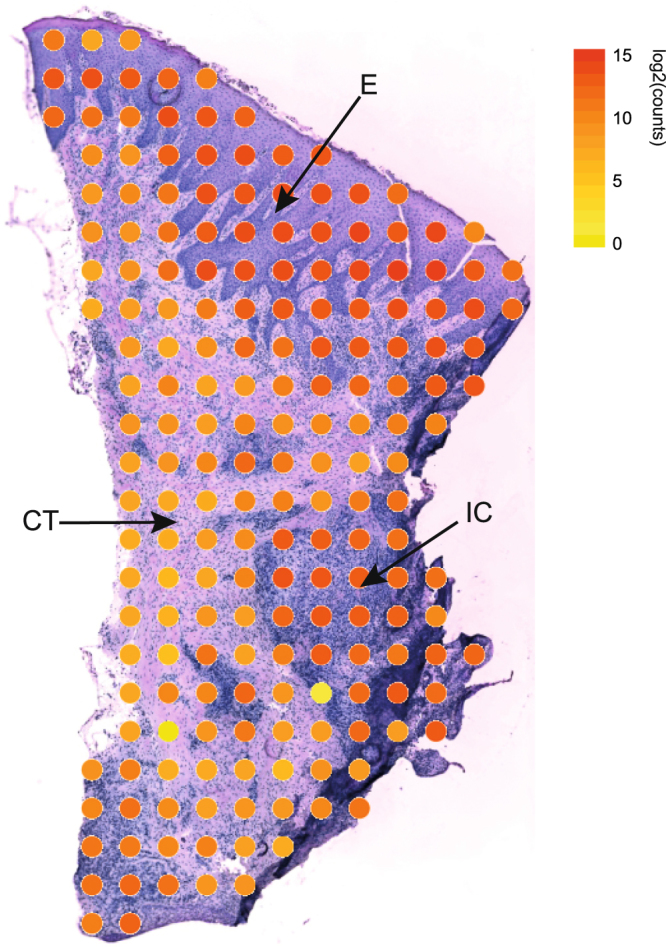
Gene activity within periodontitis-affected gingival tissue. The total numbers of transcripts per spot are visualized at their corresponding location within the gingival biopsy section. The spots are colored according to their log2 transformed total transcript content (yellow = low number of transcripts, red = high number of transcripts). CT: connective tissue; E: epithelium; IC: inflammatory cells.

To investigate the gene activity in the gingival tissue section, the amount of transcripts detected in each spot was visualized in the context of their localization within the tissue. Our results, visualized in Fig. 1, revealed that the gene activity was highest in the epithelium compartment and in the areas of connective tissue with infiltrating inflammatory cells. The gene activity was lowest in areas of the connective tissue without inflammatory cells (Fig. 1). In total, the analysis identified 15,965 unique genes expressed within the gingival tissue section.

### Specific gene expression signatures within the gingival tissue

**Figure 2:**
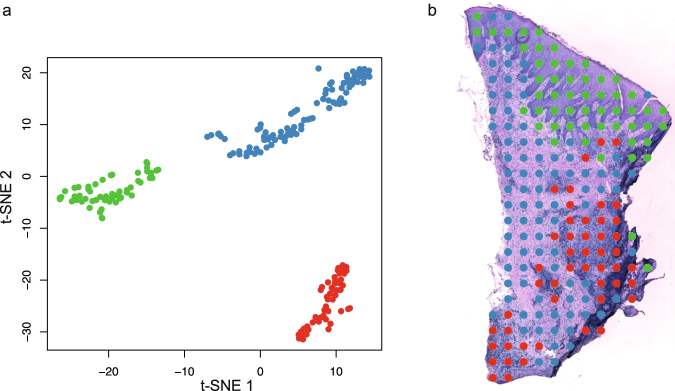
Dimensionality reduction of the gene expression data obtained from the periodontal biopsy section. (**a**) t-distributed stochastic neighbor embedding (t-SNE) plot performed on the gene expression data from spots covered by tissue. K-means clustering analysis has identified three groups of spots, which have been assigned a color each. (**b**) The spots visualized at their original positions in the gingival tissue. The colors indicate the clusters each spot was assigned to in the t-SNE plot in (**a**).

To explore gene expression profiles within the tissue section, the dimension reduction technique t-distributed stochastic neighbor embedding (t-SNE) was applied to the obtained gene expression data. Three distinct signatures of gene expression could be identified by the t-SNE analysis as demonstrated in Fig. 2a. Each dot in the t-SNE projection represents the gene expression of a single spot in the spatial transcriptomics array. To identify the corresponding tissue of each dot, the dots were first color-coded according to their clustering in the t-SNE and thereafter visualized according to their coordinates on the spatial transcriptomics array and overlaid an image of the tissue. The results of the analysis revealed that the three gene expression signatures were attributed to three distinct regions in the tissue section; the epithelium, areas in the connective tissue with infiltrating inflammatory cells, and areas in the connective tissue without infiltrating inflammatory cells (Fig. 2b).

### Gene expression in inflamed and non-inflamed connective tissue

**Figure 3:**
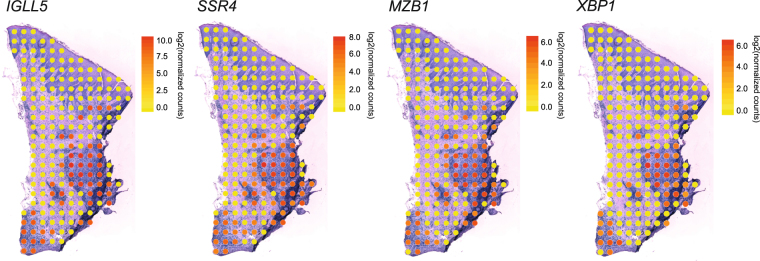
Distribution of gene activity of the four genes up-regulated to the greatest extent in areas of inflammatory cells compared to areas without inflammatory cells. The gene expression distribution of immunoglobulin lambda-like polypeptide 5 (*IGLL5*), signal sequence receptor subunit 4 (*SSR4*), marginal zone B and B1 cell specific protein (*MZB1*) and X-box binding protein 1 (*XBP1*) are shown. Yellow spots correspond to low expression of *IGLL5*, *SSR4*, *MZB1*, or *XBP1*, whereas red spots correspond to high gene expression. All count values have been normalized and log2 transformed.

In order to investigate genes differentially expressed between inflamed and non-inflamed areas of the connective tissue obtained from a patient with periodontitis, gene expression from spots covered by connective tissue with infiltrating inflammatory cells were compared to gene expression from spots covered by connective tissue without inflammatory cells. Differential expression analysis revealed ninety-two genes that were up-regulated, as well as three genes that were down-regulated, in inflamed areas as compared to the non-inflamed areas. The ten most highly up-regulated genes, as well as the three down-regulated genes are demonstrated in Table 1. The four genes up-regulated to the greatest extent in the areas of inflammatory cells, compared to the areas without inflammatory cells, were immunoglobulin lambda-like polypeptide 5 (*IGLL5*), signal sequence receptor subunit 4 (*SSR4*), marginal zone B and B1 cell specific protein (*MZB1*) and X-box binding protein 1 (*XBP1*). Distributions of the gene activities of the top four genes were visualized on top of the tissue image as demonstrated in Fig. 3, which confirmed elevated expression of these three genes in areas of the connective tissue with inflammatory cells, compared to connective tissue without infiltrated inflammatory cells.

**Table 1:** The most highly up- and down-regulated genes in inflamed regions of the gingival connective tissue.

Ensembl ID	Gene symbol	Gene name	Log2 fold change	Adjusted p-value
**Up-regulated genes**				
ENSG00000254709	*IGLL5*	Immunoglobulin lambda-like polypeptide 5	11.5	3.0E-12
ENSG00000180879	*SSR4*	Signal sequence receptor subunit 4	8.7	4.1E-7
ENSG00000170476	*MZB1*	Marginal zone B and B1 cell specific protein	7.6	1.8E-5
ENSG00000100219	*XBP1*	X-box binding protein 1	7.5	2.3E-5
ENSG00000105369	*CD79A*	Cluster of differentiation 79A	7.3	3.9E-5
ENSG00000166710	*B2M*	Beta-2 microglobulin	6.9	1.0E-4
ENSG00000099958	*DERL3*	Derlin 3	6.7	1.5E-4
ENSG00000142534	*RPS11*	Ribosomal protein S11	6.7	1.3E-4
ENSG00000234745	*HLA-B*	Major histocompatibility complex, class I, B	6.7	1.3E-4
ENSG00000179218	*CALR*	Calretucilin	6.6	1.3E-4
**Down-regulated genes**				
ENSG00000205076	*LGALS7*	Galectin 7	−7.0	0.03
ENSG00000106624	*AEBP1*	Ae binding protein 1	−2.4	0.04
ENSG00000108821	*COL1A1*	Collagen type I alpha 1 chain	−2.1	0.02

The ten most highly up-regulated genes, as well as the three down-regulated genes in areas of the gingival connective tissue with infiltrated inflammatory cells compared to areas without inflammatory cells, are demonstrated. The Ensembl ID, gene symbol, gene name, log2 fold change and Benjamini-Hochberg adjusted p-value are reported for each gene.

The biological processes of the up-regulated genes were further investigated (Table 2). The three most significantly enriched categories in the inflamed areas compared to the non-inflamed areas were SRP-dependent cotranslational protein targeting to membrane (GO:0006614), antigen processing and presentation of peptide antigen via MHC class I (GO:0002474), and antigen processing and presentation of exogenous peptide antigen via MHC independent (GO:0002480). The ten most significantly enriched gene ontology categories among the up-regulated genes are demonstrated in Table 2.

**Table 2:** The top ten most significantly enriched biological processes in areas of inflammatory cells.

GO term	Biological process	Fold enrichment	Adjusted p-value
GO:0006614	SRP-dependent cotranslational protein targeting to membrane	19.8	1.34E-6
GO:0002474	Antigen processing and presentation of peptide antigen via MHC class I	43.5	4.17E-6
GO:0002480	Antigen processing and presentation of exogenous peptide antigen via MHC class I, TAP-independent	103.7	2.52E-5
GO:0034976	Response to endoplasmic reticulum stress	19.9	2.85E-5
GO:0000184	Nuclear-transcribed mRNA catabolic process, nonsense-mediated decay	14.1	3.65E-5
GO:0006413	Translational initiation	12.3	8.98E-5
GO:0036498	IRE1-mediated unfolded protein response	22.1	7.99E-5
GO:0043066	Negative regulation of apoptotic process	5.7	8.03E-5
GO:0019083	Viral transcription	13.3	1.97E-4
GO:0006412	Translation	7.4	6.42E-4

The ten gene ontology (GO) categories, with regard to biological processes, that were identified as most significantly associated with genes up-regulated in inflamed areas compared to non-inflamed gingival connective tissue. GO term, biological process, fold enrichment and Benjamini-Hochberg adjusted p-value are demonstrated.

### Validation of up-regulated genes and their protein products

**Figure 4:**
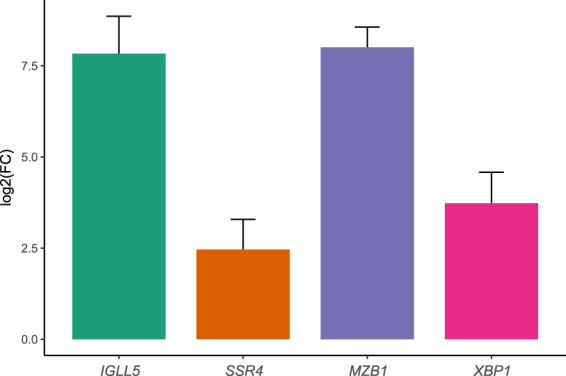
Gene expression validation in gingival tissue biopsies using qRT-PCR. The four most highly upregulated genes in connective tissue with inflammatory cells, compared to connective tissue without inflammatory cells, as identified by spatial transcriptomics, were validated using qRT-PCR in RNA from six gingival tissues from patients with periodontitis and four control samples without periodontitis. Gene expression is shown as log2 fold change (FC) of periodontitis samples relative to healthy controls and was calculated according to the ΔΔCt method, where each periodontitis sample was normalized to the control samples and to *GAPDH* (reference gene) of corresponding periodontitis sample. The reactions were run in duplicates and experiment was repeated with similar results. *IGLL5*: immunoglobulin lambda-like polypeptide 5; *SSR4*: signal sequence receptor subunit 4; *MZB1*: marginal zone B and B1 cell specific protein; *XBP1*: X-box binding protein 1.

In order to validate genes as differentially expressed between inflamed and non-inflamed areas of the connective tissue, we also investigated gene expression levels in gingival biopsies obtained from patients with periodontitis and healthy controls using quantitative reverse transcription polymerase chain reaction (qRT-PCR). The levels of the four most highly up-regulated genes, *IGLL5*, *SSR4*, *MZB1* and *XBP1*, were investigated in gingival RNA samples from ten subjects (six with periodontitis and four healthy controls). All four genes were significantly up-regulated (p < 0.05) in the gingival biopsies from patients with periodontitis compared to controls (Fig. 4).

**Figure 5:**
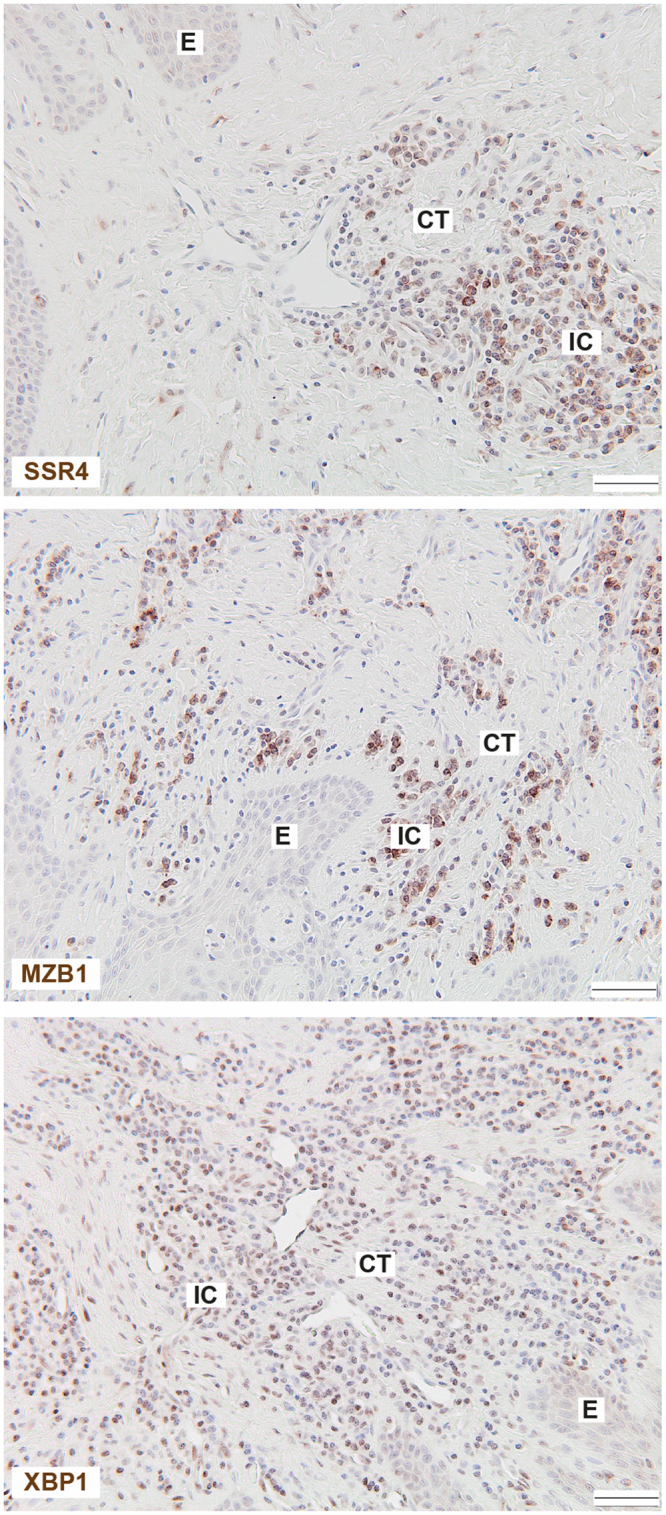
Protein expression in gingival tissue biopsies by immunohistochemistry. Gingival tissue sections obtained from patients with periodontitis were stained for signal sequence receptor subunit 4 (SSR4), marginal zone B and B1 cell specific protein (MZB1) and X-box binding protein 1 (XBP1). The sections were counterstained with Mayer’s hematoxylin. Images were taken with x20 objective. Representative images of gingival biopsies from four patients with periodontitis are shown. Scale bar: 50 μm. CT: connective tissue; E: epithelium; IC: inflammatory cells.

Furthermore, we explored the protein expression of the most highly up-regulated genes, using immunohistochemistry. The immunohistochemical analysis confirmed that SSR4, MZB1 and XBP1 proteins were expressed in gingival tissue from patients with periodontitis (Fig. 5). The proteins SSR4 and XBP1 were observed in both epithelium and connective tissue, whereas MZB1 localized mainly in connective tissue in the areas with inflammatory cells. The protein product for IGLL5 could not be validated because specific antibodies were not commercially available. Control stainings with the isotype-matched irrelevant antibodies were negative (figure not shown).

## Discussion

Periodontitis is a complex disease with multifactorial etiology^18^. In order to identify significant genes for periodontitis, we have previously investigated the gene expression profiles in gingival tissue biopsies from patients with periodontitis and healthy controls, using RNA-seq. In this study we report, for the first time, the simultaneous quantification and localization of gene expression in periodontitis-affected gingival tissue.

First, we investigated the level of gene expression in the tissue section, revealing highest gene expression in the epithelium, the second highest gene expression in areas with infiltrated inflammatory cells within the connective tissue and low gene expression in the connective tissue without inflammatory cell infiltration. The higher amount of transcripts in the epithelium is likely due to the higher density of cells as compared to the more sparsely distributed cells of the connective tissue. Thereafter, we investigated whether there were specific patterns of gene expression within the tissue section, using the dimension reduction technique t-SNE. This analysis revealed three distinct clusters of gene expression, which could be attributed to the epithelium, areas of inflammatory cells in the connective tissue, and non-inflamed areas in the connective tissue. This implies that not only the level of gene activity, but also the expressions of specific genes differ between these areas.

Next, we examined the difference in gene expression between the inflamed and non-inflamed regions within the connective tissue compartment. Our analysis revealed 442 genes that were up-regulated in inflamed areas, whereas no genes could be identified as up-regulated in non-inflamed areas. One explanation for this could be due to the fact that genes constitutively expressed by the cells of the connective tissue are expressed both in inflamed and non-inflamed regions. However, in response to inflammation, the inflamed regions have an up-regulation of genes expressed by infiltrated inflammatory and resident cells, in addition to the genes constitutively expressed by the connective tissue.

The four most highly up-regulated genes in the inflamed areas, compared to the non-inflamed areas, were *IGLL5*, *SSR4*, *MZB1* and *XBP1*. The genes *IGLL5* and *MZB1* have previously been reported as up-regulated in gingival tissue from patients with aggressive or chronic periodontitis, compared to healthy controls^19,20^. However, little is known about the function of the *IGLL5* gene, but it is homologous to *IGLL1*, which is reported to be involved in B-cell development^21^. The MZB1 protein regulates surface presentation and secretion of IgMs^22–24^. Regarding *SSR4*, the gene encodes the delta subunit of the translocon-associated protein subunit (TRAP), which is involved in translocating proteins across the endoplasmic reticulum membrane^25–27^. In addition, there are indications that *SSR4* is involved in beta cell survival in type 2 diabetes, but this gene has however, to our knowledge, not previously been reported as involved in periodontitis-affected gingival tissue^28^. The fourth most up-regulated gene, *XBP1*, is a transcription factor reported to be necessary for differentiation of plasma cells and its protein expression is required for the transcription of class II major histocompatibility genes^29,30^. The most highly up-regulated genes described above were also validated using RT-qPCR and immunohistochemistry, in a separate cohort of gingival tissue samples obtained from patients with periodontitis and healthy subjects, confirming gene expression results obtained by spatial transcriptomics.

The most highly enriched gene ontology terms associated with the genes up-regulated in the inflamed area were related to functions associated with localization and targeting to the ER, which is responsible for the folding, processing, and trafficking of secreted and membrane-bound proteins, which includes many key components of the immune response^31^. Up-regulation of genes related to protein production and control may be due to an overall larger activity in the inflamed area than in the non-inflamed area.

The drawback with standard RNA-seq is that it is performed on whole biopsies and it is not possible to localize the gene expression within the biopsy without *post-hoc* tests such as fluorescent *in situ* hybridization, or investigating the genes’ protein products using immunohistochemistry, which are low-throughput techniques in comparison to RNA-seq. The strength of the current study is that we can simultaneously quantify and localize gene expression within gingival tissue in a high-throughput manner.

In summary, in this study we report distinct gene expression signatures within periodontitis-affected gingival tissue, as well as specific genes that are up-regulated in inflamed areas compared to non-inflamed areas of gingival tissue using the novel technology spatial transcriptomics. The results obtained from this study may improve our understanding of the genes and cell types contributing to the chronic inflammatory disease periodontitis.

## Methods

### Ethics approval

This study was performed in accordance with the Declaration of Helsinki and current Swedish legislation. The gingival tissue collection was approved by the Regional Ethics Board in Stockholm with reference number 2008/1935 – 31/3 and written informed consent was obtained from all individuals included in the study.

### Gingival tissue collection

Gingival tissue biopsies were collected from subjects with periodontitis and healthy controls without periodontitis as previously described^17,32^. The inclusion criteria for periodontitis comprised tooth sites with probing depth ≥6 mm, clinical attachment level ≥5 mm, and bleeding on probing. Healthy control subjects showed no signs of periodontal disease, with no gingival inflammation, a probing depth ≤3.0 mm, a clinical attachment level ≤3.5 mm, and no bleeding on probing^17^.

### Generation of sequencing libraries

Sequencing libraries were prepared as previously described^33,34^. Briefly, the gingival biopsies were snap-frozen, embedded in OCT and sectioned on a cryostat at a thickness of 10 μm. Six sections were selected and mounted on a glass slide where barcoded probes had been deposited in 1,007 spots in an array-format. The tissue sections were fixed with formaldehyde, stained with haematoxylin and eosin, and imaged under a microscope. For subsequent analysis, the section of the tissue with most intact morphology was chosen. Following permeabilization of the tissue section, reverse transcription was performed *in situ* with the probes attached on the glass slide functioning as primers, resulting in complementary DNA (cDNA) coupled to the barcoded probes on the array. The tissue was thereafter enzymatically digested and removed from the glass slide, after which the probes were enzymatically cleaved. The barcoded cDNA was collected and libraries were generated, which were sequenced on the Illumina NextSeq platform using paired-end sequencing.

### Sequence alignment and generation of gene expression database

For each pair of reads, one read contained the positional barcode and the other contained the gene transcript. The read containing the positional barcode was converted into its corresponding x and y coordinates on the array, whereas the read containing the transcript was aligned against the human genome. The gene alignment and x and y coordinates were added to a database and this database was used for all following analyses. Detailed information on sequence processing is available in a recently published study^34^.

### Statistical analysis and visualization of gene expression data

A series of analyses investigating the gene expression together with the spatial information was conducted. All analyses were performed using R software, version 3.3.3^35^, if not otherwise specified. The data was filtered to only contain gene expression from spots covered by tissue. For analyses associating gene expression with morphology, spots were plotted according to their x and y coordinates and overlaid on an image of the tissue section, so that the spots appeared on their original positions in the tissue section.

First, the total amount of gene transcripts in each spot was visualized by plotting the spots color-coded according to their total transcript content (yellow to red corresponding to low to high transcript content). For this analysis, log2 transformed gene expression data was used. In the next analysis, a truncated singular value decomposition (SVD) was applied to the gene expression data, followed by t-distributed stochastic neighbor embedding (t-SNE), using the scikit-learn package in python^36^. The t-SNE algorithm was employed since it is widely used for single-cell RNA-seq data analysis^37–41^, which is more similar to our spatial transcriptomics data than is standard RNA-seq data. In addition, this dimension reduction technique has previously been reported to model spatial transcriptomics RNA-seq data well^34^. The truncated SVD decomposed the gene expression data into ten components, which together explained 98.19% of the total variance, and these components were used as input for the t-SNE. Three clusters of gene expression patterns were readily visible after constructing a t-SNE plot, and k-means clustering was run to assign the spots to three groups. The groups were assigned a color each and the spots were thereafter plotted and overlaid onto the image of the tissue section, as described above.

Differential gene expression analysis was performed between spots covered by inflamed connective tissue, and spots covered by non-inflamed tissue, using DESeq2^42^, with a modified zero-tolerant computation of the geometric mean and an adjusted p-value cutoff of 0.05. For this analysis, an additional adjacent section from the same biopsy was included. Moreover, due to that the inflamed connective tissue had markedly higher transcript content per spot than the non-inflamed areas, the transcript counts were summed for the spots in the non-inflamed connective tissue in each section. In the DESeq2 analysis, the two summed groups of spots from the non-inflamed areas were treated as two samples, and all spots in the inflamed areas were considered a sample each. The distribution of gene expression for the three most highly up-regulated genes were visualized at the corresponding locations in the tissue section. Log2 transformed normalized counts (as obtained from the DESeq2 analysis) was plotted for each gene, color-coded yellow to red, corresponding to low to high gene expression.

Gene ontology category analysis was performed on the differentially expressed genes using the Database for Annotation, Visualization and Integrated Discovery (DAVID) tool^43^ with the “GOTERM_BP_ALL” option selected.

### Quantitative RT-qPCR

Total RNA was isolated from gingival tissue biopsies of ten subjects with and without periodontitis (six patients with periodontitis and four healthy controls) as previously described^17^ using the RNeasy Mini Kit (Qiagen, Valencia, CA, USA). The amount of total RNA was quantified using a Qubit spectrophotometer (Molecular Probes, Eugene, Oregon, USA). cDNA synthesis was performed from 1 µg of total RNA per 20 µl of reaction using the iScript™ cDNA Synthesis Kit (BioRad, Herkules, CA, USA), according to the manufacturer’s instructions. The gene expression analysis was performed by quantitative reverse transcription PCR (qRT-PCR) using TaqMan Gene Expression Assays together with TaqMan Universal PCR Master Mix (Applied Biosystems, Foster City, CA, USA). TaqMan probes were used as follows: *IGLL5* (Hs04330879_u1), *SSR4* (Hs00196721_m1), *MZB1* (Hs00414907_m1), *XBP1* (Hs00231936_m1) and the housekeeping gene glyceraldehyde 3-phosphate dehydrogenase (*GAPDH*; Hs02758991_g1). All qRT-PCR reactions were run in duplicates on the 7500 Fast Real-Time PCR system (Applied Biosystems). Gene expression was calculated according to the ΔΔCt method, where each periodontitis sample was normalized to the healthy control sample and to *GAPDH* (reference gene) of corresponding sample. Statistical significance was calculated using Wilcoxon signed rank test and p < 0.05 was considered significant.

### Immunohistochemistry staining

Gingival tissue samples were fixed and embedded, following standard procedures as previously described^17^. For immunohistochemistry experiments, the sections (4 μm thick) were deparaffinised and antigen retrieval was performed using citrate buffer, pH 6.0. The R&D Systems Cell and Tissue Staining Kit (CTS005) was used for the blocking steps, secondary antibodies and chromogenic staining. Primary antibodies for SSR4 (1:200, Atlas Antibodies, Stockholm, Sweden), MZB1 (1:500, Atlas Antibodies, Stockholm, Sweden), and XBP1 (1:500, Abcam, Cambridge, UK) were applied overnight. All washing steps and incubations with primary antibodies were performed in PBS with 0.1% saponin. The sections were counterstained with Mayer’s hematoxylin (Histolab Products AB, Gothenburg, Sweden), dehydrated and mounted with PERTEX® (Histolab Products AB, Gothenburg, Sweden). All steps were carried out at room temperature. As negative isotype control polyclonal rabbit IgG (0.0005 ug/ml, DAKO, Agilent Technologies, Santa Clara, CA, USA) was used.
